# Genetic Sex and Origin Identification Suggests Differential Migration of Male and Female Atlantic Bluefin Tuna (*Thunnus thynnus*) in the Northeast Atlantic

**DOI:** 10.1111/eva.70009

**Published:** 2024-09-19

**Authors:** Einar Eg Nielsen, Kim Birnie‐Gauvin, Henrik Baktoft, Haritz Arrizabalaga, Tomas Brodin, Massimiliano Cardinale, Michele Casini, Gustav Helström, Teunis Jansen, Anders Koed, Petter Lundberg, Brian R. MacKenzie, Antonio Medina, Søren Post, Naiara Rodriguez‐Ezpeleta, Andreas Sundelöf, José Luis Varela, Kim Aarestrup

**Affiliations:** ^1^ National Institute of Aquatic Resources Technical University of Denmark Silkeborg Denmark; ^2^ AZTI, Marine Research Basque Research and Technology Alliance (BRTA), Herrera Kaia Pasaia Gipuzkoa Spain; ^3^ Department of Wildlife, Fish, and Environmental Studies Swedish University of Agricultural Sciences Umeå Sweden; ^4^ Department of Aquatic Resources, Institute of Marine Research Swedish University of Agricultural Sciences Lysekil Sweden; ^5^ Department of Biological, Geological and Environmental Sciences University of Bologna Bologna Italy; ^6^ GINR – Greenland Institute of Natural Resources Nuuk Greenland; ^7^ Departamento de Biología, Facultad de Ciencias del mar y Ambientales Universidad de Cádiz Cádiz Spain; ^8^ AZTI, Marine Research Basque Research and Technology Alliance (BRTA) Sukarrieta Bizkaia Spain

**Keywords:** ecological genetics, fisheries management, population ecology, population genetics ‐ empirical

## Abstract

Knowledge about sex‐specific difference in life‐history traits—like growth, mortality, or behavior—is of key importance for management and conservation as these parameters are essential for predictive modeling of population sustainability. We applied a newly developed molecular sex identification method, in combination with a SNP (single nucleotide polymorphism) panel for inferring the population of origin, for more than 300 large Atlantic bluefin tuna (ABFT) collected over several years from newly reclaimed feeding grounds in the Northeast Atlantic. The vast majority (95%) of individuals were genetically assigned to the eastern Atlantic population, which migrates between spawning grounds in the Mediterranean and feeding grounds in the Northeast Atlantic. We found a consistent pattern of a male bias among the eastern Atlantic individuals, with a 4‐year mean of 63% males (59%–65%). Males were most prominent within the smallest (< 230 cm) and largest (> 250 cm) length classes, while the sex ratio was close to 1:1 for intermediate sizes (230–250 cm). The results from this new, widely applicable, and noninvasive approach suggests differential occupancy or migration timing of ABFT males and females, which cannot be explained alone by sex‐specific differences in growth. Our findings are corroborated by previous traditional studies of sex ratios in dead ABFT from the Atlantic, the Mediterranean, and the Gulf of Mexico. In concert with observed differences in growth and mortality rates between the sexes, these findings should be recognized in order to sustainably manage the resource, maintain productivity, and conserve diversity within the species.

## 1 Introduction

Across the animal kingdom, key aspects of the biology of species differ between sexes including morphology, growth, natural mortality, maturation, and migration (De Lisle 2019). This is primarily caused by the phenomenon of anisogamy—the different sizes of gametes of males and females (Parker, Baker, and Smith 1972)—leading to different selective pressures on the two sexes (Bateman 1948). Thus, knowledge about the sex of individuals is of key importance for understanding the evolution, life history, and ecology of a species, as well as for assuring proper management and conservation. For example, if males and females differ in their habitat selection and migration patterns (Ohms et al. 2019), they could experience differential exploitation patterns (see Bade et al. 2019 and references therein), which could have negative consequences on the population's growth and sustainability, when not properly accounted for in assessment models. This would be the case even if one sex is not specifically targeted (Stubberud et al. 2019). Likewise, overexploitation of one sex can skew the operational sex ratio at reproduction, thereby leading to a decline in the genetically effective population size and reduce genetic variability (Coltman 2008).

For many species, visual sex identification in the field is relatively straightforward due to highly distinct primary and/or secondary sexual characters; however, for other species including mammals (Bartolommei et al. 2015), birds (Griffiths 2000), and fish (Suda et al. 2019), there is little or lacking sexual dimorphism, particularly for immature individuals. Thus, sex determination is often invasive, requiring extensive handling or, as is common for fish, is achieved through direct but lethal inspection of gonads. Accordingly, focus of ecological research has shifted towards noninvasive sex determination, including DNA‐based applications, which are accurate as well as time‐ and cost‐efficient (Hrovatin and Kunej 2018; Suda et al. 2019).

The Atlantic bluefin tuna (ABFT), *Thunnus thynnus*, is an iconic species of huge commercial and recreational value (McKinney et al. 2020). It lacks clear sexual dimorphism, thereby hampering inferences on differences in biology and ecology between males and females for live animals. This is especially relevant within the context of the extensive pop‐up satellite archival tagging (PSAT) programs conducted on both sides of the Atlantic, which have gained exclusive new information on migration routes, oceanographic residency and preferences, spawning and feeding areas, and life‐history features such as skipped spawning (e.g., Wilson et al. 2015; Cermeño et al. 2015; Horton et al. 2020; Aarestrup et al. 2022). Hitherto, migration and other ecological data have not been disaggregated into male and female components to elucidate sex‐specific patterns, which may prove crucial for interpreting observed differences among geographical and temporal ABFT aggregations. Until recently, no method was available for sexing BFT noninvasively; however, whole genome sequencing has identified sex‐specific regions in Pacific bluefin tuna (PBFT), *T. orientalis* (Suda et al. 2019; Nakamura et al. 2021), and assays for male‐specific markers for routine sexing have been developed (Suda et al. 2019). Given the close evolutionary proximity between ABFT and PBFT (Díaz‐Arce et al. 2016), it is highly likely that they share a common XY sex determination system, which pre‐dates speciation. Moreover, the male‐specific marker assays have been shown to be readily transferrable between tuna species, including ABFT (Chiba et al. 2021).

In recent years, ABFT has seen a positive stock development in the Northeast Atlantic (ICCAT 2023). This has been associated with an increased distributional area, including the re‐emerging of summer feeding aggregations in North Atlantic waters, such as around the British Isles (Horton et al. 2020), Scandinavia (Nøttestad, Boge, and Ferter 2020; Aarestrup et al. 2022), and even in Greenlandic waters (Jansen et al. 2021). Population genetic analysis has shown that the stock is primarily made up of individuals originating from the Mediterranean spawning population, but with a low inferred proportion of individuals from the Gulf of Mexico population (Rodríguez‐Ezpeleta et al. 2019; Jansen et al. 2021). As these newly claimed feeding areas of the Eastern Atlantic population distribution are subject to emerging recreational and commercial fisheries, information on sex ratio and population of origin is crucial to inform stock assessment models that aim to secure stock productivity and genetic variability for long‐term sustainable management. At the same time, extensive electronic tagging projects are ongoing in the region (e.g., Aarestrup et al. 2022), where information on sex and genetic population of origin is essential for understanding the mechanisms that drive individual behavior.

Here, we use the transferability of the PBFT genetic sexing assays to ABFT by genotyping individuals of known sex from aquaculture. We apply a modified simple genetic single‐tube reaction assay for sexing 296 samples of ABFT collected from 2017 (Aarestrup et al. 2022) to 2020 in Scandinavian waters (Kattegat/Skagerrak) and 33 samples from East Greenland (Jansen et al. 2021). In addition, all samples were genotyped using a panel of 96 single nucleotide polymorphisms (SNPs) (Rodríguez‐Ezpeleta et al. 2019) to determine if they belong to either Eastern Atlantic (Mediterranean) or Western Atlantic (Gulf of Mexico) spawning populations. The aims were to investigate basic features of newly established ABFT feeding aggregations in Northern regions by determining: (1) the relative occurrence of individuals from the two main spawning populations, (2) whether sex ratios are male or female biased, (3) if sex ratios vary across years, geographical areas, and in relation to population of origin, and (4) the presence of a relationship between sex and size (fork length).

## 2 Materials and Methods

### 2.1 Sampling

**FIGURE 1:**
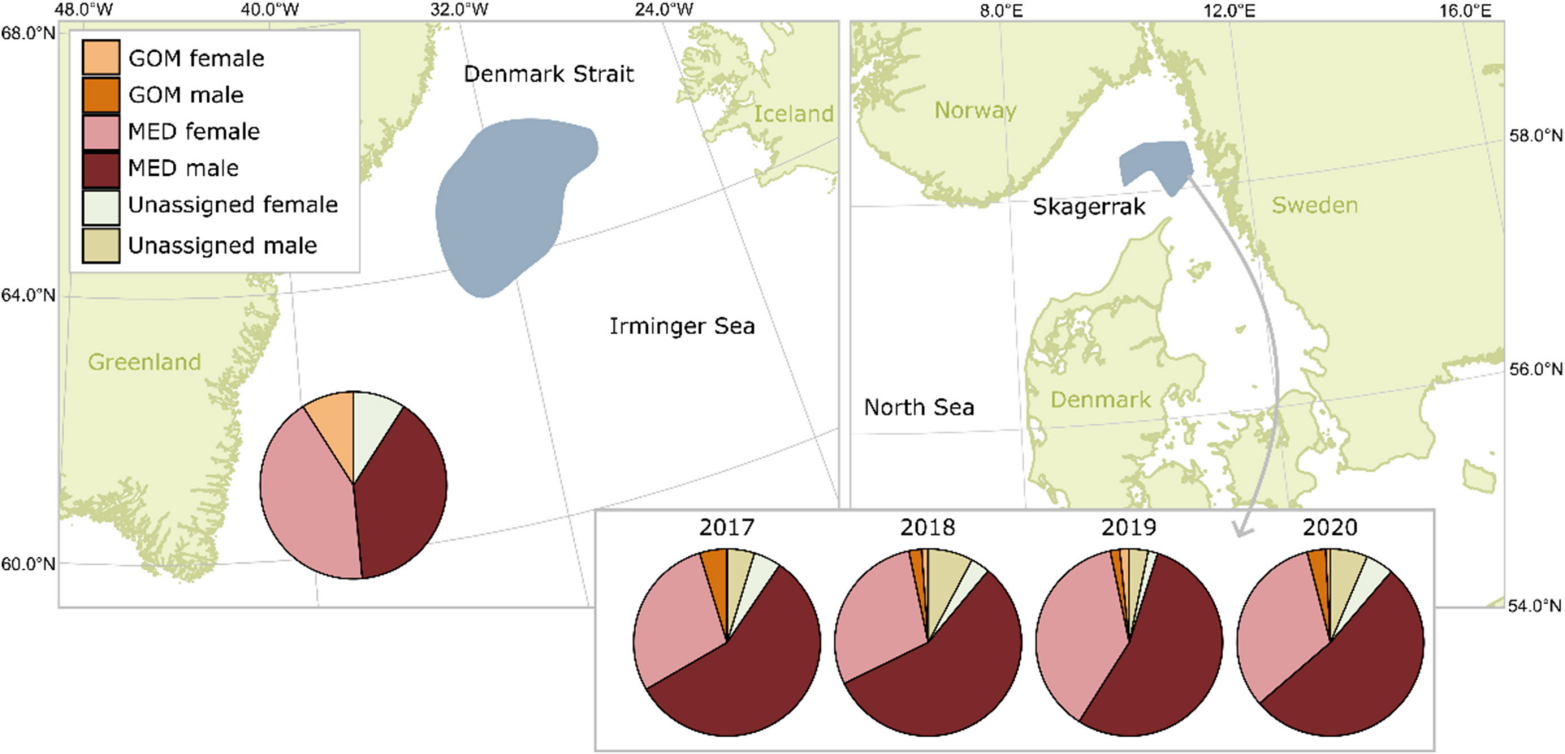
Approximate sampling locations and proportions of male and female ABFT assigned to eastern Atlantic (MED), western Atlantic (GOM), and unassigned. Left: Eastern Greenland. Right: The Skagerrak (see text for explanation).

The primary sample collection of ABFT genetic samples was conducted in the Skagerrak among Denmark, Sweden, and Norway (see Figure 1, right), where 296 tissue samples (fin clips) were collected between 2017 and 2020 (*n* = 21, 90, 61, and 124, respectively) in August and September. The sampled ABFT were captured by volunteer big game anglers using rod‐and‐reel as part of a large‐scale tagging project including PSATs. Of the 296 sampled specimens, 282 (95%) had length measurements (curved fork length). In addition, 33 ABFT muscle samples were analyzed, which were collected from bycatch specimens from the East Greenland mackerel fishery (2014, 2015, and 2017). All individuals were caught within a rectangle covering the area between 64° N, 34° W; 64° N, 30° W; 66° N, 34° W; 66° N, 30° W (Figure 1, left). Finally, six tissue samples from ABFT of known sex were retrieved from a collection of adult mature individuals, which had been visually sexed based on gonadal examination (A. Medina, personal communication).

### 2.2 Development and Test of Sex Identification Assays

Primer pairs I and II originally developed for PCR‐based sex identification in PBFT (Suda et al. 2019) were used. Briefly, they found a 6.5 kb region containing 44 male‐specific SNPs on the designated scaffold_064. The targeted primers specifically amplified segments within the largest linkage disequilibrium block (3174 bp). Primer pairs were initially tested on the ABFT individuals of known sex (three of each sex, see above). In addition, we included a positive amplification marker (microsatellite Tth 04, Clark, Saillant, and Gold 2004) to ensure that nonamplifying individuals were not mistakenly identified as females.

### 2.3 Population Genetic Analysis

The genetic stock identification panel developed by Rodríguez‐Ezpeleta et al. (2019), consisting of 96 discriminatory SNPs was used for assigning ABFT back to the genetic populations from the two main spawning grounds: Gulf of Mexico (GOM) and Mediterranean Sea (MED). We applied an 80% probability criterion for individual fish, where the misassignment rates between the two populations have previously been shown to be 2% for individuals of known MED origin, and 10% for individuals with known GOM origin (consisting of larvae, young of the year and spawning adults from GOM and MED respectively, Rodríguez‐Ezpeleta et al. 2019). The genetic analyses were conducted as described in Jansen et al. (2021). Briefly, DNA was extracted from muscle samples using the E.Z.N.A. kit (Omega Biotek, Norcross, Georgia, USA) and SNPs were genotyped using the Biomark HD platform (Fluidigm) and 96.96 Dynamic Array IFCs. Assignment was conducted with the GeneClass2 software (Piry et al. 2004) using a minimum probability score criterion of > 80% for positively inferring population of origin. Individuals with lower probabilities were classified as “unassigned”.

### 2.4 Statistical Analyses

Probability of individual ABFT at a given length being female or male was analyzed using a Bernoulli distributed generalized additive model (GAM) using *R v.4.3.2* (R Core Team 2023) and package *mgcvv.1.9* (Wood 2011).

## 3 Results

### 3.1 Sex Identification Assays

The six visually sexed ABFT individuals all provided sex‐specific DNA amplification patterns as seen in PBFT (Suda et al. 2019), with two amplified male‐specific bands for primer sets I and II of similar size, as reported for PFBT (113 and 143). All six reference individuals provided positive amplification for the microsatellite Tth 04. The consistent amplification of the two male‐specific bands in our reference aquaculture specimen reconfirmed that the previously developed molecular assay for sex identification in PBFT (Suda et al. 2019) also works for ABFT (Chiba et al. 2021).

### 3.2 Sex Ratios and Population Affinity

Of the 33 individuals caught in Greenlandic waters, three were assigned to GOM, 27 to MED, and three were unassigned. All three GOM individuals were females. For the individuals assigned to MED, there were 14 females and 13 males, thus not significantly different from a 1:1 sex ratio. Of the 296 ABFT samples collected in Skagerrak from 2017 to 2020, 11 samples were assigned to GOM, 256 to MED, and 29 were unassigned (see Figure 1). There was no significant difference in distribution of GOM and MED assignments among the 4 years (χ^2^ = 0.21, d.f. = 3, *p* = 0.98). The individuals assigned to GOM consisted of eight males (73%) and three females (27%), while the MED assignments comprised 161 males (63%) and 95 females (37%), which was significantly different from a 1:1 sex ratio (χ^2^ = 8.65, d.f. = 1, *p* = 0.003) and varied between 35% and 41% among years (χ^2^ = 0.90, d.f. = 3, *p* = 0.83). The sex‐ratio difference between GOM and MED assigned individuals was nonsignificant (χ^2^ = 0.44, d.f. = 1, *p* = 0.51). The distribution of GOM and MED assigned individuals did not differ significantly between Greenland and Skagerrak (χ^2^ = 2.0761, d.f. = 1, *p* = 0.15). Likewise, the sex ratio for the specimen assigned to MED was not significantly different between the two areas sampled (χ^2^ = 2.24, d.f. = 1, *p* = 0.13).

### 3.3 Sex and Size

**FIGURE 2:**
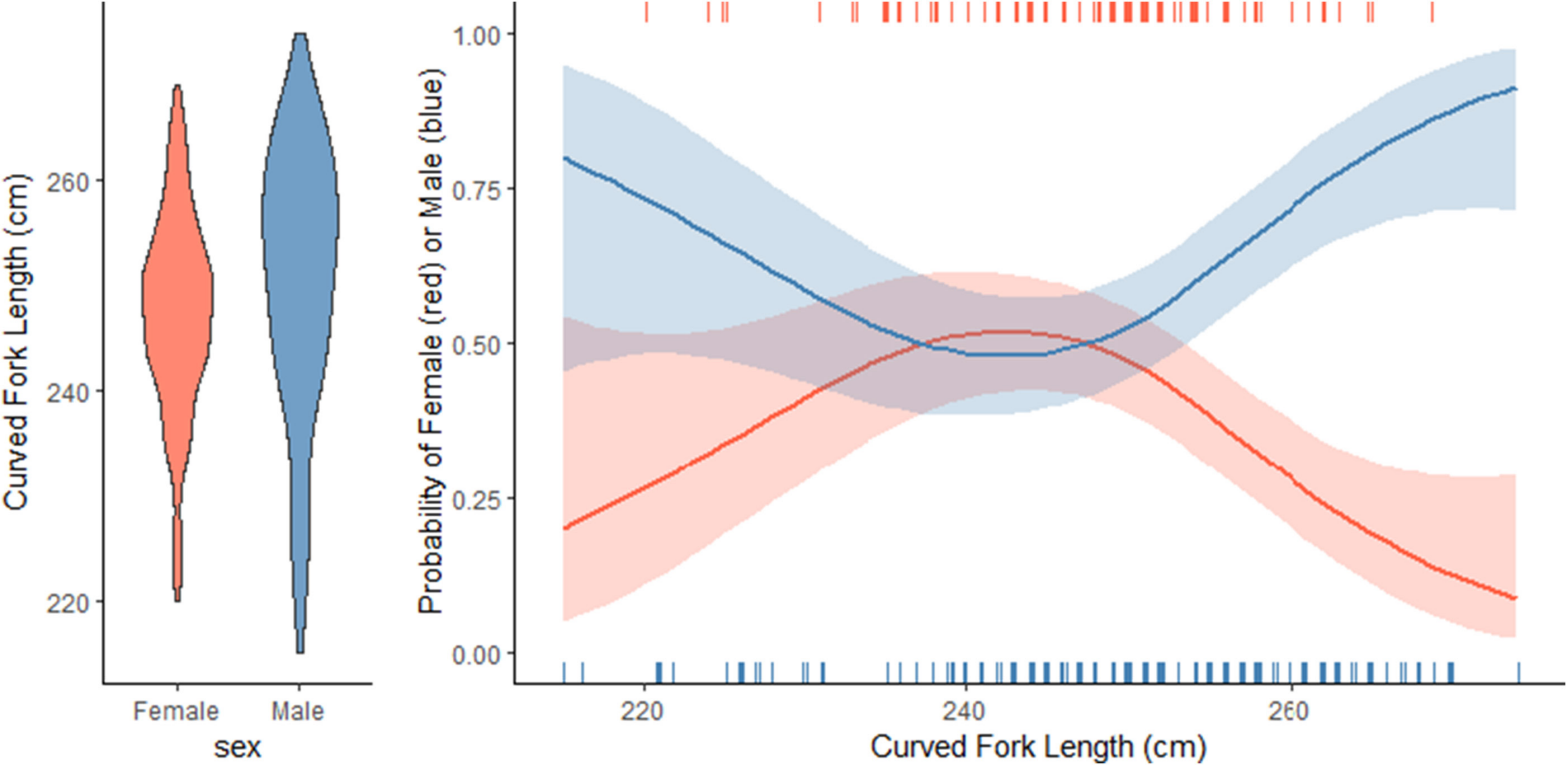
Left: Distribution of female (orange) and male (blue) ABFT lengths for individuals sampled in the Skagerrak that assigned to the eastern Atlantic (MED) population. Right: Probability for observing males (blue) and females (orange) at different lengths. Curves represent model‐predicted mean values and 95% confidence intervals. Vertical ticks show raw data.

For the Skagerrak samples, the mean size of individuals measured, genotyped, and positively assigned to population (*n* = 251) was 249.0 cm (SD ±12.4 cm). For individuals assigned to MED, the mean size of males (*n* = 146) was 250.4 cm (SD ±12.8 cm), while for females (*n* = 94) it was 246.7 cm (SD ±11.3 cm). For individuals assigned to GOM, the mean size of males and females was 254.8 cm (SD ±8.6 cm, *n* = 8) and 240.7 (SD ±6.8 cm, *n* = 3), respectively. For the MED individuals sampled in Skagerrak, the distribution of sizes was different between the two sexes (Figure 2, left), with most females at intermediate sizes (240–259 cm) and most males at larger sizes (250–274 cm). Most of the small individuals (185–229 cm) were males. This, in concert with the generally higher proportion of males, resulted in a pronounced higher probability for observing males at small and large sizes (Figure 2, right), but with a close to equal probability at intermediate sizes (GAM model: d.f. = 3.08; Δdeviance = 21.8, *p* = 7.7E−5).

## 4 Discussion

### 4.1 Population Affinity

Samples from both locations within the Northeast Atlantic—East Greenland and the Skagerrak—consisted almost exclusively of individuals assigned to the Eastern Atlantic (MED) population (90% and 96%, respectively). The proportion of Western Atlantic (GOM) individuals is so low that we cannot reject the hypothesis that they occur due to mis‐assignment alone, as their contribution is not significantly higher than the observed mis‐assignment rate of the method (2%), evaluated by using 165 eastern Atlantic reference samples (Rodríguez‐Ezpeleta et al. 2019). Thus, all individuals sampled, genotyped, and assigned could originate from the eastern Atlantic population. However, the occurrence of individuals of western Atlantic origin in the eastern Atlantic has been inferred previously using genetics (Rodríguez‐Ezpeleta et al. 2019) and microchemistry (Rooker et al. 2019). The data support the previous observation (Rodríguez‐Ezpeleta et al. 2019) that individuals closer to the most northeasterly part of the ABFT feeding distribution in the Northeast Atlantic have a relatively low proportion of western migrants. The proportion of unassigned individuals (10%) is also similar to the rates reported by using panels of genetic markers for origin assignment (Puncher et al. 2022; Rodríguez‐Ezpeleta et al. 2019). The relatively high rate of unassigned individuals compared to other marine fish species, which has used genomic information to develop SNP panels for origin assignment (Nielsen et al. 2012; Jenkins, Ellis, and Stevens 2019; Farrell et al. 2022) can be ascribed to the shallow genetic population structure of the species, but potentially also to the recent finding of a new spawning area in the Slope Sea situated between the northeast United States Continental Shelf and the Gulf Stream (Richardson et al. 2016; Hernández et al. 2022). Here, hybridization between the ancestral eastern and western populations apparently occurs (Díaz‐Arce et al. 2024), suggested to be caused by the recent expansion of the eastern population and related intensification of westward migration. This results in intermediate genotypes, which by default cannot be assigned to any of the ancestral populations of origin with high certainty. Consequently, the unassigned individuals could, at least partly, consist of individuals originating from the admixed population in the Slope Sea. The relative recruitment and demographic connectivity of the Slope Sea population compared to the ancestral populations is unknown (Dias‐Arce et al. 2023); thus, the potential significance of Slope Sea individuals in our samples remains elusive. Still, our samples consist almost exclusively of individuals assigned to the eastern population with high statistical certainty; thus, the occurrence of Slope Sea individuals is unlikely to seriously affect the main conclusions of this study.

### 4.2 Sex Ratio

There was a clear male bias in the individuals sampled with inferred Eastern Atlantic origin. Although the sex ratio among individuals from Eastern Greenland was approximately 1:1, it did not differ significantly from the sex ratio observed in the Skagerrak samples, due to the relatively low sample size for the East Greenland samples and associated low statistical power. For the individuals assigned to the western population, the sex ratio was also male biased for the Skagerrak samples but not for the East Greenland samples, where all three individuals assigned to the western Atlantic population were females. Interestingly, the three unassigned individuals from east Greenland were all females assigned to the western population, that is, with a statistical power above 50% but below the set 80% threshold. Thus, although we cannot reject that the sex ratios differed due to low statistical power, our data suggest that there could be a female bias for the western Atlantic, or possibly Slope Sea, individuals occurring in Eastern Greenland. More sexed and population‐assigned individuals are required to test this hypothesis.

Several factors could be responsible for the observed male bias. For many species displaying partial/differential migration (Chapman, Jørgensen, and Lutcavage 2011; Secor 2015), the propensity to migrate can be caused by a “conditional strategy” (Lundberg 1988) either due to extrinsic state such as environmental conditions or behavioral dominance, or by intrinsic factors such as age, sex, and condition factor. Consequently, it has been shown, also among fish species, that males and females commonly have different propensities to migrate (Secor 2015; Ohms et al. 2019). Thus, it is possible that male ABFT, due to genetic or environmental factors (or a combination), are predisposed for a higher migration rate or longer (northward) migration distances. Generally, females tend to be the migratory sex in fish (Ohms et al. 2019), related to the consequences of anisogamy, that is, that females generally invest more resources in gamete production (Hayward and Gillooly 2011) and, therefore, will benefit more from risking predation during feeding migration. Alternatively, intense mating competition among males could also favor a larger size (Kim et al. 2021) and associated migration benefits. Moreover, differences in habitat suitability, for example, in relation to size (see Druon et al. 2016 and discussion below) or differences in timing of maturity/spawning migration (Medina et al. 2022) between sexes could result in different marine occupancy for males and females. This was already suggested for large western ABFT in the Gulf of Mexico by Baglin Jr et al. (1982), who found a higher occurrence of females in spawning aggregations during April and May, and more males in feeding schools during July and August: “These findings suggest that some giant bluefin tuna segregate into distinct areal groups according to the predominating sex and that sex ratios may change with season” (Baglin Jr et al. 1982). This pattern is also found in large migratory bluefin tuna in the eastern Atlantic, where Medina et al. (2022) recently found a clear female bias for individuals <230 cm caught in a tuna trap at Cadiz (southern Spain). However, Addis et al. (2016) found a consistent overrepresentation of males in catches from a similar trap in Sardinia. Thus, there is ample evidence to suggest the presence of sex‐related differential migration and occupancy in ABFT.

The observed male bias could also be affected by differential exploitation of the two sexes. Sex‐biased fishing has been documented in many fish species including Atlantic cod (*Gadus morhua*; Robichaud and Rose 2003), Pacific halibut (*Hippoglossus stenolepis*; Loher et al. 2016) and sockeye salmon (*Oncorhynchus nerka*; Kendall and Quinn 2012). The bias is often due to indirect effects of size‐selective fishing due to differential growth of the two sex (Uusi‐Heikkilä 2020) but can also be caused by differential migration and occupancy in relation to feeding and sexual maturation (Bade et al. 2019). Thus, different behaviors and associated exploitation can act in concert to further skew sex ratios. There is ample evidence of sex‐biased exploitation from tuna traps (De La Serna, Ortiz De Urbina, and Alot 2003; El Tawil et al. 2004; Addis et al. 2016). Most studies show a higher exploitation rate among females, particularly at lower sizes (see also discussion below), although there is also evidence of male‐biased exploitation and variable sex ratios in the catch in different areas and among years within the same areas (Caria, Boulila, and Deguara 2020). Thus, the observed male‐biased sex ratio that we observe in northern feeding aggregations could also be partially caused by differential exploitation of males and females in southern Atlantic and Mediterranean fishing areas.

### 4.3 Sex and Size

The recently reclaimed summer feeding aggregations in the Northeast Atlantic showed differential sex ratios in relation to size. Males were overrepresented in both the small‐ and large‐sized fish, while an approximately equal sex ratio was found in intermediate‐sized fish. The observation that the sex ratio changes with size is not unusual in ABFT (Medina 2020) and has been demonstrated both in the fishery for large migratory tuna in the Atlantic (De La Serna, Ortiz De Urbina, and Alot 2003; dos Santos et al. 2016; Medina et al. 2022) and in the Mediterranean (El Tawil et al. 2004; Aranda et al. 2013; Addis et al. 2016) as well as in the Gulf of Mexico (Baglin Jr et al. 1982). Most often, size‐related changes in sex ratio in ABFT have been ascribed to differential exploitation (see section above), but also to a higher inferred growth rate in males due to an alleged higher cost of reproduction for females (Hurley and Isles 1983; Aranda et al. 2013; Medina 2020). This is despite Santamaria et al. (2009) finding, no significant differences between sex‐specific growth curves for individuals up to approximately 15 years of age (length ≈240 cm). Based on the large size of the individuals in our study, and the accumulated effect of repeated spawning, differential growth between sexes could have contributed to the observed size differences between males and females in the larger size classes. Thus, the large males and females could originate from one or a few cohorts where slightly different growth trajectories caused a male sex bias in the largest size class. However, neither a higher female mortality nor a higher growth of males can fully explain the higher occurrence of males in the Skagerrak feeding assemblages. That is, differential growth and mortality cannot account for the higher frequency of males in the smaller size class and then a shift back to an equal sex ratio at intermediate sizes. Alternatively, differences in catchability due to behavioral differences between the sexes could explain the biased sex ratio. However, the general observation of biased sex ratios across areas and gear type is not in line with this hypothesis, though it cannot be ruled out. Thus, some effect of sex‐specific migration propensity or patterns seems the most parsimonious explanation for the consistently higher occurrence of males and the size differences between sexes in the Skagerrak feeding aggregations. This corroborates the hypothesis of Addis et al. (2016), who proposed that the observed sex segregation of ABFT schools during their reproductive migration in Sardinia (Mediterranean) was due to some sort of sex‐specific ecological barrier. Likewise, the very female‐biased sex ratio observed recently in large migratory ABFT caught in the Strait of Gibraltar (Medina et al. 2022) and the variable or male‐biased ratio observed in the Mediterranean (Addis et al. 2016; Caria, Boulila, and Deguara 2020) is difficult to explain without inferring spatial or temporal segregation in occupancy, migratory route, or timing between male and female ABFT.

### 4.4 Implications for Management

Information on the demography of exploited fish populations is essential for sustainable harvest and conservation of biological resources. In relation to stock assessment, information on the sex of individuals is part of the basic good practice guidelines (Punt 2023). Information on sex is generally relevant for assessing fisheries and natural mortality, as well as growth, fecundity, gear selectivity, and dispersal (Punt 2023). Ideally, stock assessments should be based on an age‐ or size‐structured population dynamic model considering differential growth, mortality, maturation, and migration for the two sexes (Punt 2023). Likewise, negligence of sex‐based behavioral differences in relation to exploitation can lead to abnormal operational sex ratios at spawning grounds, leading to reduced egg deposition or sperm limitation (Bade et al. 2019) and reduced genetically effective population size (Nunney 1993) depending on mating structure. Moreover, the specific reduction of larger individuals of one sex can lead to the disruption of mating systems and, in the long term, to evolutionary changes in the size distribution (Uusi‐Heikkilä 2020). Accordingly, international conservation policies such as the EU Marine Strategy Framework Directive (MSFD; European Union 2008) explicitly consider that population demographic characteristics (e.g., body size or age class structure, sex ratio, fecundity, and survival rates) should not be affected by anthropogenic pressures.

Our findings, in concert with previous studies, strongly suggest that male and female ABFT in the Northeast Atlantic likely differ in many ways, be it growth, fisheries mortality, natural mortality, and/or migration patterns, all of which are important for successful management. Therefore, information on sex should be a priority and a prerequisite for future assessments and adequate management of ABFT populations. This has not been done so far due to the lack of visual sex dimorphism; however, genetic sexing, in combination with information on the population of origin, would provide an ideal and informed foundation to study sex‐specific differences in behavior. Many studies have already used individual electronic tagging by applying PSATs for ABFT (Block et al. 2005; Goldsmith, Scheld, and Graves 2017; Pagniello et al. 2023), including in the recently rediscovered feeding aggregations (Aarestrup et al. 2022), but none have combined tagging with sex and population of origin. The integration of these novel methods could really improve our understanding of the biology of ABFT, with important implications for how we manage the species. Occupancy of males and females in relation to seasonal fishing pressure in various areas may result in unwanted sex‐biased fishing mortality. Thus, information may reveal when areas should be opened or closed for fishing, focusing on both maintaining a sustainable, productive, and lucrative commercial and recreational fishery, while also securing biological diversity and productivity. Besides the applicability of our method for future research and management, this study also generated new hypotheses regarding the underlying mechanisms driving sex biases in feeding aggregations.

## Ethics Statement

We declare that this study has not been published or submitted elsewhere. 44 fish (of 329) in this study had previously been genetically assigned to population without information on sex and published in Jansen et al. (2021) and Aarestrup et al. (2022). All procedures and experimental protocols were conducted in accordance with the Danish Experimental Animal Inspectorate permit for DTU (License no. 2017‐15‐0201‐01164) and approved by DTU Aquas' animal welfare committee, and in accordance with the routines of the Swedish Board of Agriculture approved by the animal ethics committee (5.8.18‐11659/2017 and 5.8.18‐08180/2018).

## Conflicts of Interest

The authors declare no conflicts of interest.

## Data Availability

The data that support the findings of this study are openly available at The Technical University of Denmark's curated data repository site https://data.dtu.dk/articles/dataset/Data_for_Genetic_sex_and_origin_of_Atlantic_Bluefin_Tuna/26762854 and can be cited as Eg Nielsen (2024). Genetic sex and origin identification suggests differential migration of male and female Atlantic bluefin tuna (*Thunnus thynnus*) in the Northeast Atlantic DOI: 10.11583/DTU.26762854.
